# A Study on Biocompatibility of Three Endodontic Sealers: Intensity and Duration of Tissue Irritation 

**Published:** 2014-03-08

**Authors:** Camilla Christian Gomes Moura, Thais Cristina Cunha, Virgínia Oliveira Crema, Paula Dechichi, João Carlos Gabrielli Biffi

**Affiliations:** a * Department of Structural Biology, Federal University of Triângulo Mineiro, Uberaba, Minas Gerais, Brazil and Department of Cell Biology, School of Dentistry, Federal University of Uberlândia, Minas Gerais, Brazil; *; b * Integrated Dental Clinic Program, School of Dentistry, Federal University of Uberlândia, Minas Gerais, Brazil; *; c * Department of Structural Biology, Federal University of Triângulo Mineiro, Uberaba, Minas Gerais, Brazil; *; d * Department of Morphology, Federal University of Uberlândia, Minas Gerais, Brazil; *; e * Department of Endodontics, School of Dentistry, Federal University of Uberlândia, Minas Gerais, Brazil*

**Keywords:** Biocompatible Materials, Biocompatibility Testing, Endodontics, Root Canal Filling Materials, Root Canal Obturation, Root Canal Sealants, Subcutaneous Tissue

## Abstract

**Introduction:** Several studies have evaluated the inflammatory reaction triggered by Epiphany (EPH), a contemporary endodontic sealer. However, they used conventional parameters, which need additional analysis to better understand the reactions induced by this sealer compared to other traditional sealers. **Methods and Materials:** The intensity and time span of tissue irritations for three endodontic sealers were assessed by inflammatory reactions, fibrous capsule measurement and mast cell counts. Tubes containing freshly mixed EPH, AH plus (AHP) and Endofill (ENF) were subcutaneously implanted into the backs of 28 Wistar rats. The side wall of the tube was used as the control. At 14, 21, 42 and 60 days, the connective tissue surrounding the implants (*n=*7) was stained for histopathological analysis. The Friedman test was applied to compare the results. The level of significance was set at 0.05. **Results:** At days 14 and 21, a significant difference among the groups was observed, with the ENF showing the worst tissue response (*P*<0.001). ENF remained the most aggressive sealer at 42 and 60 days, compared with EPH (*P*<0.05). No differences were found for the fibrous capsule thicknesses among the groups in each period. The number of mast cells per field did not show difference among the sealers at 21 and 60 days. **Conclusions:** EPH and AHP elicited similar patterns of irritation, as demonstrated by the inflammatory scores and fibrous capsule thicknesses. ENF caused the highest degree of tissue damage. The increase in mast cell counts observed during the early and late periods shows the possibility of late hypersensitivity to the test materials.

## Introduction

Apart from good physical and chemical characteristics, ideal root canal filling materials should provide biological compatibility. Endodontic sealers are not an exception as they can inadvertently extrude beyond the apical constriction and result in tissue irritation and delayed healing [1, 2]. The biocompatibility of root canal sealers may be influenced by their composition and setting [3]. In general, the toxicity of resin-based endodontic sealers is higher when the sealers are fresh. However, this effect is reduced over time [4].

Epiphany (EPH) is a resin-based sealer designed with the concept of mono block obturation which is supplemented by a self-etch primer to improve its adhesion to dentin [5-7]. Although several *in vitro* [4, 8, 9] and *in vivo* studies [5, 6, 10, 11] have evaluated the biological properties of this sealer, the results are influenced by the conditions under which the sealer was tested [1]. Studies that were conducted on EPH [1, 2, 12] whether fresh or polymerized [5, 6], and with or without its self-etched primer, have used different methods of biocompatibility assessment.

It is important to determine the inflammatory reaction triggered by EPH and compare this reaction to other traditional sealers in order to determine the ideal sealer [1]. Considering the rather late presence of mast cells in biocompatibility evaluation of EPH [10], and noting that the presence of these cells might be related to late hypersensitivity reactions to the endodontic sealers [13, 14], it is relevant to evaluate this parameter during histological analysis of the biomaterials [15, 16]. Furthermore, the presence of fibrous capsule have been described by some researches [6, 17-19], without correlations with the number of mast cells. Considering all these points, this study aims to compare the inflammatory reaction caused by Epiphany compared with reactions caused by Endofill (ENF), as an eugenol based sealer and AH plus (AHP), as an epoxy resin, in terms of fibrous capsules formation and the number of mast cells in the initial and late periods.

## Methods and Materials

This study was performed with the approval of the Committee on Ethics in Research with Animals of Federal University of Uberlândia (Approval no.: 016/09). The tested materials were manipulated in accordance with the manufacturers’ recommendations, and the tubes were filled as follows: EPH group (Epiphany, Pentron Clinical Technologies, Wallingford, CT, USA); ENF group (Endofill, Herpo Produtos Dentários Ltda, Petrópolis, RJ, Brazil); and AHP Group (AH Plus, Dentsply, De Trey, Konstanz, Germany).

For this study, 28 mature male Albinus Wistar rats weighing between 200-250 g were used. The animals were anesthetized by an intramuscular injection of 10 mg/100 g of body weight ketamine chlorhydrate (Cetamin, Syntec, São Paulo, Brazil) associated with 0.05 mg/mL xylazine (Anasedan, Agribrands do Brasil Ltda, São Paulo, SP, Brazil). The backs of the animals were shaved and disinfected with 5% iodine potassium iodide (IKI). Four separate dorsal pockets were created by blunt dissection with scissors to a depth of 20 mm to implant the materials in the subcutaneous tissue. A sterilized polyethylene tube (Embramed, São Paulo, Brazil) with 1.2 mm diameter (0.6 mm internal diameter) and 10 mm length, containing fresh sealer was placed into each pocket. The manipulated sealers were carefully put into polyethylene tubes with aid of a syringe attached to a needle compatible with the diameter of tubes, ensuring that there were no empty spaces and that the sealer did not overflow.

Considering that the contraction of the material inside the tube could result in the absence of contact between the material and tissue, one of the extremities of each tube was supplemented with sealer. The opening of the tube containing the sealer was always positioned towards the animal’s head. This face was chosen for histological analysis for all groups. The side wall of the tube was used as the control. The tissue was closed with cyanoacrylate tissue adhesive (Super Bonder, Henkel Loctite Adesivos Ltda, Itapevi, Brazil) to prevent tension and displacement of the material.

After 14 (*n=*7), 21 (*n=*7), 42 (*n=*7) and 60 (*n=*7) days, the rats were sacrificed by an anesthetic overdose and the tubes together with surrounding tissues were removed for histological analysis. The specimens were fixed in a 10% buffered formalin solution for 24 h and processed for conventional histological examination. The connective tissue adjacent to the open end of each tube was subjected to semi-serial sections of 5 μm in a longitudinal plane, passing through the opening of the polyethylene tube and including the interface between the material and the connective tissue. The sections were stained with Hematoxylin and Eosin (H&E) for histopathological analysis, Picrosirius red (PS) for the quantification of fibrosis and Toluidene Blue (TB) for the quantification of mast cells. The connective tissue response along the lateral wall outside the polyethylene tube served as the negative control for this technique.

***Histological analyses***

Histopathologic analyses were performed under a light microscope (Carl Zeiss, Oberkochen, Germany) at ×400 magnification, on the basis of the tissue responses stimulated by the tested materials and the lateral wall of the tubes (control group). Evaluation of the inflammatory reaction was carried out in three different areas of each section. An adaption of FDI criteria [20] and Campos-Pinto study [5] was used for evaluation of the H&E sections for the presence or absence of inflammatory infiltrate (polymorphonuclear cells and mononuclear cells), macrophage activity (macrophage and giant inflammatory cells), mast cells, dispersed material and necrotic tissue. A score was used to quantify the presence or absence of these events as follows; depending on these features, a grade from 1 to 4 was used to graduate the inflammatory reaction:

1. absent (-): no chronic inflammatory cells
2. slight (+): few inflammatory cells scattered in the connective tissue
3. moderate (++): a large number of inflammatory cells focally arranged
4. severe (+++): a large number of inflammatory cells diffused in connective tissue

In the sections stained with TB, the sealer/tissue interface was assessed using a standard light microscope at ×200 magnification. Two images per section were subjected to differential counting of mast cells for a total of six fields per animal for each analyzed group. The number of mast cells per field in the 42 fields analyzed in each group for each sealer, was subjected to statistical analysis. In sections stained with PS, the morphometric quantification of collagen-capsule thickness was performed in the corresponding tube opening. An image-analysis system consisting of an automatic microscope image capture camera and a computer with KS300 software (Kontron Zeiss, Germany) under polarized light filter, were used. Two different areas were measured for each section, excluding the periphery of the tubes.

**Figure 1:**
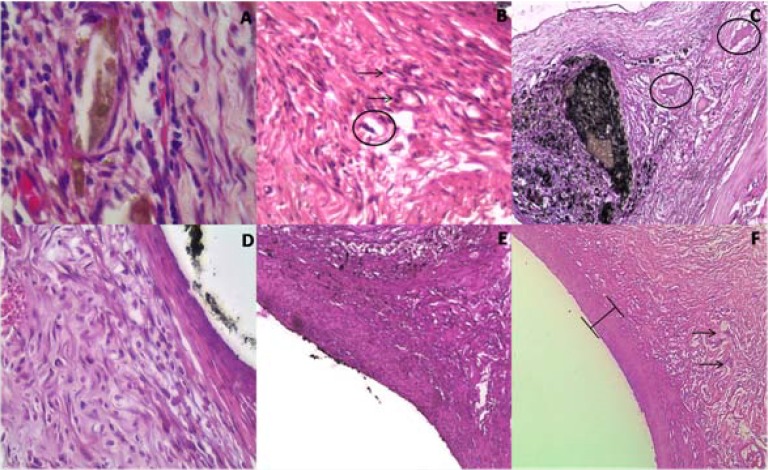
Histological reaction around Endofill (ENF), AH plus (AHP) and Epiphany (EPH), at days 14 and 21. *A)* ENF 14 days; inflammatory infiltrate with the presence of dilated blood vessels (×1000); *B)* AHP 14 days; inflammatory infiltrate, presence of blood vessels (arrow) and giant cells (circle) ×100; *C)* EPH 14 days; Region of contact with the sealer. Note the presence of foreign body giant cells (circle) ×100; *D)* ENF 21 days; inflammatory infiltrate and cellular fibrous capsule (×400); *E*) Interface AHP connective tissue at 21 days (×100); *F)* EPH 21 days; Fibrous capsule and the presence of mast cells (arrow) ×100

The final image was magnified to ×1600, with the image being shown on a monitor. The polarized image of the fibrous connective tissue showed birefringence with yellow-red coloring, and thus was quantified automatically. The results were expressed as mean percentages of collagen fibers arranged in parallel in relation to the total area per field. The observers blindly evaluated the sections. Each observer evaluated a specific parameter: histopathological analyses (H&E sections) by observer 1; mast cell counting (TB sections) by observer 2 and fibrous capsule (PS sections) by observer 3.

The results of the inflammatory reactions and comparison of the fibrous tissue thickness were tested with the Friedman, One-way analysis of variance (ANOVA) and the Kruskal-Wallis tests. The level of significance was established at 0.05.

## Results

**Qualitative analysis**

***At 14 days: ***For this period, a moderate to severe inflammatory reaction was observed for ENF, while EPH and AHP displayed a mild to moderate inflammatory infiltrate (Figure 1). Some of these specimens exhibited an initial organization of fibrous capsules. Necrosis was observed next to the dispersed material. EPH presented some areas with macrophages and hyperemic dilated blood vessels. ENF showed lymphoplasmocytic infiltration and an absence of cellular organization in the vicinity of the dispersed sealer. The control showed no inflammation characterized by a fibrous tissue.

***At 21 days: ***In this period, there was a mild to absent chronic inflammatory reaction to EPH and AHP. Macrophages were observed in areas of residual dispersed material. On the other hand, a moderate to severe inflammatory reaction close to the implant material, composed by multinucleated cells near to small foci of necrosis, was seen in the ENF specimens. In this period, the establishment of a fibrous capsule is evident. Figure 1 shows a general view of events observed at 21 and 42 days in each group.

**Figure 2:**
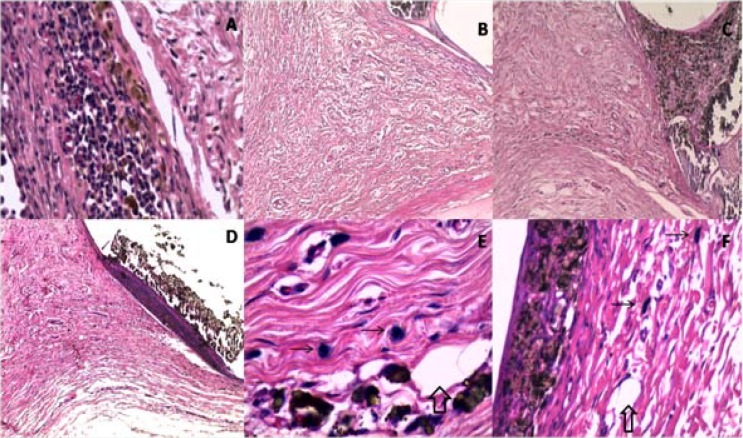
Histological reaction around Endofill (ENF), AH plus (AHP) and Epiphany (EPH), at days 42 and 60. *A)* ENF 42 days; lymphocytic infiltrate (×400); *B)* AHP 42 days; thin fibrous capsule, presence of macrophages, giant cells (×100); *C)* EPH 42 days; region in contact with the sealer limited by a fibrous capsule (arrow), note the absence of inflammation (×100); *D)* ENF 60 days; panoramic view, presence of well-defined fibrous capsule (×40); *E)* AHP 42 days; presence of blood vessels (hollow arrow) and mast cells (arrow) ×1000; *F)* EPH 60 days; presence of fibrous capsule surrounding the sealer, blood vessels (hollow arrow) and mast cells (arrow) ×400

***At 42 days: ***EPH and AHP presented histological features similar to those at 21 days with mild to absent chronic inflammatory reactions and residual dispersed materials in some areas. ENF presented a moderate to mild inflammatory reaction consisting of lymphoplasmocytic infiltration and macrophages. Figure 2 shows a general view of events observed at 42 and 60 days in each group.

***At 60 days: ***EPH and AHP groups presented none to mild chronic inflammatory reaction, without presence of foreign body giant cells or macrophages. Small areas of mild inflammatory reactions were associated with little residual dispersed sealer. ENF showed mild to moderate inflammatory reactions, though some specimens had no inflammation. This sealer exhibited a fibrous capsule with high amounts of cell and vessels in some specimens.

***Quantitative analysis***

In all analyzed periods a significant difference on biocompatibility among the groups was observed. ENF sealer was the most aggressive one (14 days, *P*=0.0003; 21 days, *P*<0.0001, 42 days, *P*=0.0272 and 60 days, *P*=0.0036).

**Figure 3:**
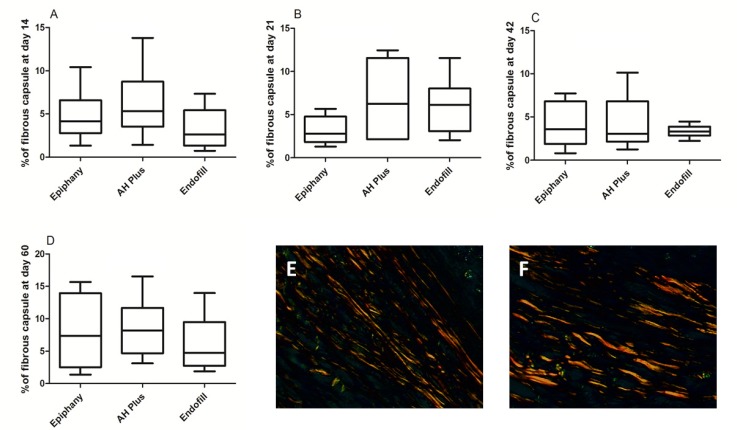
Plot Box showing median values of fibrous tissue thicknesses on Epiphany (EPH), AH plus (AHP) and Endofill (ENF) groups at days 14 ; *A)*, 21; *B)*, 42; *C)* and 60; *D)* Illustrative images of collagen fibers under polarization (Picrosirius red staining, magnification of ×400)

No significant differences were found in thickness of fibrous capsules among the groups in each time period (14 days, *P*=0.60; 21 days, *P*=0.42; 42 days, *P*=0.71; 60 days, *P*=0.65). Mean values of fibrous tissue thicknesses are shown in Figure 3. No statistical differences on fibrous capsule thickness were detected for APH (*P*=0.10) and ENF (*P*=0.60) sealers along the time. However, the area of fibrosis varied for EPH (*P*=0.003).

**Figure 4:**
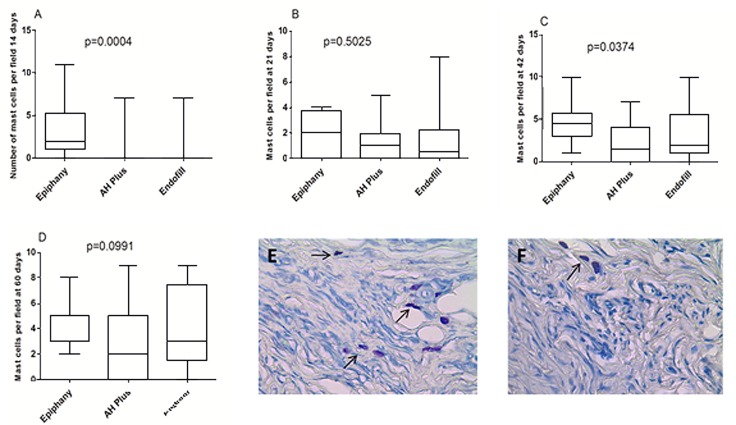
Plot Box showing median values of mast cells on Epiphany (EPH), AH plus (AHP) and Endofill (ENF) groups at days 21; *A)*, 42; *B)* and 60; *C)*. *D-E)* Illustrative images of mast cells (arrows) (Toluidene blue staining, magnification of ×200

The number of mast cells per field did not show any statistically significant difference among the sealers at 42 days (*P*=0.78) (Figure 4). The EPH group showed a higher number of mast cells per field at 14 (*P*=0.02) and 60 (*P*=0.03) days but their number got lower at 21-day period (*P*=0.001), compared with the other groups. The number of mast cell varied, depending on the time, and showed significant differences in all sealers: EPH (*P*=0.0002), ENF (*P*=0.06) and AHP group (*P*=0.02).

## Discussion

This study evaluated the inflammatory reactions generated by the first generation of EPH compared with two traditional sealers; AHP and ENF. Although some researchers have investigated physical [20, 21] and biological properties of EPH [1], its comparison to other contemporary materials helps to form a critical view about the potential biological benefits of using this material. AHP was chosen for comparison because of its wide use and low toxicity observed *in vitro* [21] and *in vivo *[22]. The results of studies using animal models cannot be directly extrapolated to actual practice, as they do not provide clinically relevant information on the long-term tissue response [13]. Along this line, the current study used an implantation model as a secondary test for evaluation of the local toxicity of endodontic sealers. Although the FDI technical report recommends the assessment of biocompatibility of dental materials in bone cavities, it is known that the primary contact of extruded endodontic sealer or its released products happens in periapical tissues, which justifies the use of subcutaneous implants in several researches of sealer biocompatibility [6, 14]. Polyethylene tubes were used as implantation vehicles because of their inert nature and suitability for putting the test material into contact with connective tissues [12], which was confirmed by the absence of an inflammatory reaction in the sidewall of the tube after 14 days. According to Silva-Herzog *et al.*, the 14-day period should be considered as the initial period of the analysis, during which it is possible to determine the irritating potential of the sealers in fresh conditions, with minimal influence from the operatory trauma [23].

The results observed at 14 days are supported by the current literature [1]. The authors did not find differences in the tissue inflammatory response between EPH and AHP sealers after 15 days. However, these results differ from the one that observed moderate to severe reactions for EPH and AHP during the same period [12]. Differences in that study compared with the present research might be attributed to the criteria used for the histopathological analysis. The inflammatory tissue response in that study was assessed on the basis of the number of inflammatory cells. However, in traditional stain techniques, that method has a limited ability to identify the types of the present cells [2]. Furthermore, quantitative assessment of inflammatory cells is possible only when there is a great difference between the inflammatory reaction generated by the study materials [1, 19], which is not true for EPH and AHP.

On the other hand, ENF, a zinc oxide-eugenol sealer (ZOE), promoted a moderate to severe inflammatory reaction up to 21 days, which was more intense than that produced by AHP and EPH. Other studies have reported a prolonged irritating effect for ZOE sealers [1, 2, 12, 19, 24]. However, in the present research, the inflammatory response decreased over time in this group. The difference in the intensity of the inflammatory reaction in the late periods (42 and 60 days) in this study compared with previous researches might be attributable to the amount of material used and post-implant time [19, 24]. The powder/liquid ratio on ZOE-based sealers might also be related to tissue irritation [24]. Previous studies attributed the low tissue tolerance of this sealer to the slow and prolonged release of eugenol [25], which is associated with the deleterious potential of zinc ions [26].

In later periods, the EPH and AHP groups have shown a marked reduction in the intensity of inflammatory reactions, ranging from mild to absent. Though both sealers trigger a similar tissue response, without statistical differences in all analyzed periods, the connective tissue in contact with EPH presented a more organized appearance at days 42 and 60. These subtle differences between AHP and EPH may explain the results observed at days 42 and 60 when both sealers were compared to ENF. Though EPH and AHP are resin sealers, they have different bases, which may result in specific tissue reactions, depending on the contact of leachable substances to the surrounding tissue [10, 12]. In general, fresh resin-based sealers exert some toxic effects, and these effects decrease over time as the concentration of leachable components is reduced [3].

The present study also evaluated the thickness of the fibrous capsule in the interface tissue/material. Previous studies have used this parameter in implantation tests performed with biomaterials [6, 17, 19]. It has been postulated that the characteristics and thickness of the fibrous capsule around the sealers may indicate if the material was tolerated by adjacent tissues [13]. In the present research, there were no significant differences for fibrous tissue thicknesses between the evaluated sealers at all analyzed periods, though qualitative analysis showed a thicker fibrous capsule in the regions where there was some extrusion of the sealer. The absence of statistical significance between the groups can be explained by differences in the thickness of the capsule in different regions of the same section. The present findings differ from previous research, which showed differences in the thickness of the capsule depending on the sealer’s composition and analysis time [13, 19]. This divergence can be justified by the difference in the parameters of the measurement used.

Mast cell analysis must take into account a possible relationship between these cells and late hypersensitivity reactions to the composition of implanted materials [13, 14]. However, the presence of mast cells in all experimental periods led to rejection of this hypothesis. Another hypothesis was raised: the presence of mast cells in late periods are related to the thickness of the fibrous capsule. Previous studies have demonstrated the relation between the presence of fibrosis and the number of mast cells in skin [27] and tongue [28]. Mast cells are enrolled in the genesis of connective tissue, releasing mediators that stimulate collagen synthesis [27, 29], which may be supported by the present study, since the number of the mast cells fluctuate along the time.

The data from this study, are in line with the study by Berbert *et al.* which indicates a new way to interpret the presence of mast cells in histopathological analysis of root canal sealers [15]. The set of presented data indicates that EPH and AHP are less aggressive in early periods after implantation than ENF, though the three sealers could be considered biologically acceptable as they show a reduced inflammatory response over time, which induces the formation of a fibrous capsule.

## Conclusion

In conclusion, within the limitations of this *in vivo* study, EPH and AHP elicited a similar pattern of irritation and connective tissue response as measured by inflammatory reaction scores and fibrous capsule thickness. ENF caused the highest degree of tissue damage, which decreased after 21 days. None of the tested materials seem to be related to the development of late hypersensitivity reaction as the number of the mast cells varied in early and late periods. All the tested sealers are biocompatible, but significantly, the best results belonged to EPH and AHP after 14 days. Therefore, both these materials might be classified as less aggressive to the tissues and more suitable endodontic sealers.
